# Bioactive Peptides

**DOI:** 10.3390/foods6050032

**Published:** 2017-04-26

**Authors:** Eric Banan-Mwine Daliri, Deog H. Oh, Byong H. Lee

**Affiliations:** 1 Department of Food Science and Biotechnology, Kangwon National University, Chuncheon 24341, Korea; ericdaliri@yahoo.com (E.B.-M.D.); deoghwa@kangwon.ac.kr (D.H.O.); 2 Department of Microbiology/Immunology, McGill University, Montreal, QC H3A 0G4, Canada

**Keywords:** functional foods, antihypertensive peptides, bioactivity, cytotoxicity

## Abstract

The increased consumer awareness of the health promoting effects of functional foods and nutraceuticals is the driving force of the functional food and nutraceutical market. Bioactive peptides are known for their high tissue affinity, specificity and efficiency in promoting health. For this reason, the search for food-derived bioactive peptides has increased exponentially. Over the years, many potential bioactive peptides from food have been documented; yet, obstacles such as the need to establish optimal conditions for industrial scale production and the absence of well-designed clinical trials to provide robust evidence for proving health claims continue to exist. Other important factors such as the possibility of allergenicity, cytotoxicity and the stability of the peptides during gastrointestinal digestion would need to be addressed. This review discusses our current knowledge on the health effects of food-derived bioactive peptides, their processing methods and challenges in their development.

## 1. Introduction

Proteins in foods do not only serve as nutrients but also perform physiochemical roles that promote health. Most of the physiological activities of proteins are performed by peptide sequences encrypted in the parent protein which become active when cleaved intact [1]. Bioactive peptides are released during enzymatic proteolysis (gastrointestinal digestion, in vitro hydrolysis using proteolytic enzymes) of proteins and also during food processing (cooking, fermentation, ripening). Bioactive peptides are known for their ability to inhibit protein-protein interactions due to their small size and specificity. Nature remains the largest source of bioactive peptides since plants, animals, fungi, microbes and their products contain various proteins in them. Over the years, bioactive peptides in foods have been discovered through a classical approach or a bioinformatic approach. The classical approach involves hydrolyzing proteins with food-grade proteolytic enzymes to release numerous peptide fragments in the hydrolysate [2,3,4]. Alternatively, proteins could be fermented by bacteria [5,6,7]. The bacteria proteolytic enzymes hydrolyze the proteins to release peptides into the hydrolysate. The hydrolysates are then tested in vitro for a biological activity. If the hydrolysates show good bioactivity, they are then confirmed through in vivo testing. The biologically active hydrolysate could then be developed into a functional food. The bioactive peptides in the hydrolysates could also be separated and purified into nutraceuticals for nonpharmacological therapy.

On the other hand, the bioinformatic (in silico) approach relies on information available on a database to determine the frequency of occurrence of already identified bioactive peptides in a protein of interest. Specific enzymes that can cleave the identified segments from the parent protein are chosen to hydrolyze the peptides. This strategy enhances the identification of known peptides from unknown proteins. A key challenge in bioactive peptide development for therapeutic purposes has however been the difficulty in establishing a cause and effect relationship between bioactive peptide consumption and their intended health effects in humans. Yet, since studies continue to confirm the therapeutic effects of bioactive peptides, a comprehensive review of recent advances in bioactive peptide research is warranted. In this review, we discuss the recent advances in the production of bioactive peptides from food including antidiabetic peptides, cholesterol-lowering peptides, antihypertensive peptides, anticancer peptides, antimicrobial peptides and multifunctional peptides. We also discuss the effects of processing methods on the bioactivity of peptides and the challenges associated with bioactive peptide development.

## 2. Production and Processing of Food Protein-Derived Bioactive Peptides

From literature, the most common methods to produce bioactive peptides have been by enzyme hydrolysis of food proteins or by fermentation [8]. In few situations however, water extracts of mushrooms and some plant parts have been found to contain bioactive peptides [9].

### 2.1. Enzymatic Hydrolysis

In this process, the protein material is subjected to enzyme hydrolysis at a given temperature and pH [10,11,12,13]. The use of enzymatic hydrolysis to produce bioactive peptides is preferred than microbial fermentation due to the short reaction time, ease of scalability and predictability. More than a single proteolytic enzyme (whether purified or crude) can be used to hydrolyze the protein to produce the hydrolysate containing short peptide sequences. However, addition of the enzymes (whether simultaneously or sequentially) would depend on the optimal pH and temperature of the enzymes [14,15,16,17]. Though no specific proteolytic enzymes are known to produce specific bioactive peptides in foods, subtilisin hydrolysis tends to yield low molecular weight peptides some of which are bioactive. For instance, Huang et al. [18] found that subtilisin hydrolyzed *Achatina fulica* snail foot muscle protein had a higher number of small molecular weight peptides than samples hydrolyzed by papain and trypsin. Zhang et al. [19] also showed that subtilisin hydrolysed rice bran proteins generated the highest number of low molecular weight peptides and showed higher biological activity than samples hydrolyzed with a cysteine endopeptidase, papain and pepsin. However, the enzyme to substrate ratio is an important factor to consider so as to obtain a good degree of hydrolysis. Peptide sequences and their biological activities may differ depending on the type of enzyme used [20]. Low molecular weight peptides (<10 kDa) have been found to be more effective antioxidants and antihypertensive peptides [21,22,23,24] than high molecular weight peptides and hence proteases that yield low molecular weight peptides would be helpful for commercial production of antioxidant and antihypertensive peptides.

Since foods may contain several other non-protein bioactive compounds, it is advisable to separate such compounds from the food proteins to avoid interference. For instance, phenolic compounds are known for their antioxidant [25], antihypertensive, antidiabetic [26] and antimicrobial [27] abilities and hence, when present in hydrolysates, can interfere with the biological activities being assayed. Phenolic compounds could be separated from food proteins using methods such as ethanol extraction [24], supercritical carbon dioxide [28] pressurized water extraction [29], ultrasound-assisted extraction [30] and acetone extraction [31] prior to enzymatic hydrolysis.

Protons released during proteolysis result in fluctuations in the pH of the medium and this may affect the efficiency of the hydrolytic process. Though the pH can be adjusted by the addition of acid or alkali solutions [13,32], addition of alkali usually results in high salt concentrations in the hydrolysates. It is therefore advisable to perform the proteolysis in a buffer [24,33,34]. The type of enzyme used, the temperature and the time allowed for hydrolysis affect the extent of hydrolysis and may also affect the type of peptides generated in the hydrolysates. For instance, Rerri et al. [35] observed stronger anti-tyrosinase and anti-inflammatory activities after hydrolyzing rice derived proteins with bacillolysin than samples hydrolyzed with subtilisin while cysteine endopeptidase, papain and leucyl aminopeptidase hydrolyzed samples showed the lowest bioactivity. They also found that, bacillolysin hydrolysed samples had the strongest anti-angiotensin converting enzyme (ACE) activity followed by cysteine endopeptidase, and then subtilisin. However, other studies found that chymotrypsin or cysteine endopeptidase hydrolyzed rice proteins had strong antioxidant abilities [36,37].

After enzymatic hydrolysis, the mixture is centrifuged to separate the supernatant which contains low molecular weight peptides from the precipitates [32,38,39]. The peptides may be recovered by freeze-drying, desalting [19], cross-flow membrane filtration [35], and membrane ultrafiltration or column chromatography. Gel filtration can be used to quickly desalt low molecular weight peptides and separate them based on their sizes.

### 2.2. Microbial Fermentation

This involves culturing some bacteria or yeast on protein substrates to hydrolyze the proteins with their enzymes as they grow. The growing bacteria or yeast secret their proteolytic enzymes into the protein material to release peptides from the parent proteins. Usually, the bacterium of choice is grown to its exponential phase in a broth at a temperature suitable for the bacterial growth. The cells are then harvested, washed and suspended in sterile distilled water (usually containing glucose) and used as a starter to inoculate a sterilized protein substrate [7,40]. The extent of hydrolysis would depend on the strain used, the type of protein and the fermentation time. We observed that whey fermented by *Lactobacillus brevis* had a stronger ACE inhibitory ability than those fermented with *Lb. acidophilus, Lb. bifermentan, Lb. casei, Lb. helveticus, Lb. lactis, Lb. paracasei, Lb. plantarum and Lb. reuteri* [38]. This shows that the functionality of protein hydrolysates may differ between cultures since microorganisms have different proteolytic systems [41]. Similar results were reported by El-Fattah et al. [42] when they observed that 14 commercial dairy starters exhibited different degrees of proteolysis, ACE inhibition and antioxidant activities after milk fermentation. Bacteria of the same species may also differ in their proteolytic capacities which may result in different bioactivities. For instance, Sanjukta et al. [43] observed that fermenting soybean protein with *B. subtilis* MTCC5480 resulted in a higher degree of hydrolysis and free amino acids than samples fermented with *B. subtilis* MTCC1747. Chen et al. [44] also reported that skim milk fermented with 37 different *Lactobacillus helveticus* strains showed different extents of hydrolysis, titrable acidity, free amino nitrogen and ACE inhibitory abilities. 

Apart from bacteria starter, yeast [6,21,45,46] and filamentous fungus [47,48,49] have also been used in producing bioactive peptides. Proteins can be co-cultured using a combination of different bacteria or even yeast and bacteria to accelerate the proteolytic process [50]. After fermentation, the mixture is centrifuged and the supernatant recovered. The supernatant may then be subjected to further hydrolysis using proteolytic enzymes to obtain shorter peptide sequences [3]. Alternatively, the low molecular weight peptides in the supernatant can be recovered by solvent extraction or other methods, purified and their amino acid sequences determined by mass spectrometry.

## 3. Food-Derived Bioactive Peptides and Human Health

### 3.1. Antidiabetic Peptides

Diabetes is a metabolic disease characterized by increased blood sugar level due to insufficiencies in insulin secretion, action, or both. The disease is classified into type I and type II. Type I diabetes (insulin dependent diabetes) is an autoimmune disease that causes the beta cells of the pancreas to secrete little or no insulin. In type 2 diabetes mellitus (T2DM), however, there is an imbalance in insulin secretion and blood sugar absorption [51]. Current synthetic antidiabetic drugs may result in risks of hypoglycemia, weight gain [52], high background risk of pancreatitis [53] and gastrointestinal side effects [54] while some patients may not even tolerate them [55]. For these reasons, the search for food derived anti-diabetic peptides (Table 1) is on the increase. Such alternatives may be safe as they are from food sources and have been consumed over the years without side effects [56]. Many fermented foods such as fermented soybean contain low molecular weight peptides some of which have been shown to induce insulin-stimulated glucose uptake in 3T3-L1 cells [57] and antagonize PPAR-γ activities. The peptides AKSPLF, ATNPLF, FEELN, and LSVSVL isolated from black bean protein hydrolysates effectively inhibited glucose transporter 2 (GLUT2) and sodium-dependent glucose transporter 1 (SGLT1) [58] to reduce blood glucose levels. In another study, peptides in salmon frame protein hydrolysates were found to significantly improve glucose uptake in L6 muscle cells showing their potential to improve blood glucose uptake [59]. Similarly, LPIIDI and APGPAGP from Silver carp (*Hypophthalmichthys molitrix* Val.) protein hydrolysates showed strong competitive/non-competitive mixed-type inhibition against DPP-IV [60].

α-Amylase inhibitory peptides such as FFRSKLLSNGAAAAKGALLPQYW (CSP1), RCMAFLLSNGAAAAQQLLPQYW (CSP2) and RPAQPNYPYTAVLVFRH (CSP3) obtained from cumin seeds prevented dietary starch absorption by inhibiting the breakdown of complex starches into simpler ones [99] and may therefore have antidiabetic effects. CSP1 directly interacted with α-amylase binding site while CSP2 bound to the enzyme surface. CSP3 however bound to the enzyme interface to inhibit the enzyme activity. Huang and Wu [100] isolated an 8 kDa peptide from shark liver which significantly reduced fasting blood glucose level and caused a significant increase in hepatic glycogen levels in streptozotocin induced diabetic mice. Although many food-derived biopeptides have shown antidiabetic activities in vitro, animal studies are very limited.

### 3.2. Cholesterol-Lowering

Our bodies require healthy levels of cholesterol for the production of vitamin D and steroid hormones as well as bile acids. Yet, excess cholesterol in the blood could form plaques in arteries resulting in arteriosclerosis. Cholesterol plaques in the coronary artery could reduce oxygen supply to the heart and lead to cardiovascular diseases. Chemical agents for lowering blood cholesterol may result in liver injury or failure, myopathy [101] and diabetes [102,103] whereas other people do not tolerate statins [104]. Therefore, the search for bioactive peptides with cholesterol lowering ability has increased over the years (Table 1).

Cumin seed derived peptides CSP1, CSP 2 and CSP3 have been shown to inhibit cholesterol micelle formation, inhibit lipase activity and bind strongly to bile acids and may therefore lower cholesterol when consumed [105]. Sericin-derived oligopeptides suppressed serum total cholesterol and non-high density lipoprotein (HDL) cholesterol levels in rats fed with high-cholesterol diet. The peptides reduced cholesterol solubility in lipid micelles, and inhibited cholesterol uptake in monolayer Caco-2 cells. They also bound tightly to taurocholate, deoxytaurocholate, and glycodeoxycholate which could lead to a reduced cholesterol absorption in the gut [106]. Soybean peptides LPYP, IAVPGEVA and IAVPTGVA have been reported to effectively activate the LDLR-SREBP 2 pathway and improve LDL uptake. The peptides also inhibited HMGCoA reductase activity in HepG2 cells [107]. Likewise, the consumption of 30 g/mL of lupin protein decreased plasma proprotein convertase subtilisin/kexin type 9 levels in patients with moderate hypercholesterolaemea. The hydrolyzed lupin proteins were found to inhibit HMGCoA reductase activity in HepG2 cells and this may account for its significant hypocholesterolaemic effect [108]. In a similar way, peptides in cowpea inhibited HMGCoA reductase and reduced cholesterol micellar solubilization in vitro [109]. Peptides in rice bran protein hydrolysates were also found to inhibit cholesterol micellar solubilization and may be important in reducing cholesterol [19]. In a recent study, Hernandez et al. [110] observed that black bean and cowpea derived peptide YAAAT could tightly bind to the *N*-terminal domain of Niemann-Pick C1 (NPC1L1) to disrupt interactions between NPC1L1 and membrane proteins to enhance cholesterol absorption. Duranti et al. [111] also observed that consumption of the α′ subunit reduced plasma cholesterol levels by 36% and also upregulated liver β-very low density lipoprotein cholesterol receptors in rats. Very little is known about the effects of specific food derived peptides on reducing cholesterol levels in vivo and hence more studies are needed in this area.

### 3.3. Antihypertensive Peptides

Hypertension (high blood pressure) is characterized by a persistent systolic blood pressure (BP) value of ≥140 mmHg and a diastolic pressure of ≥90 mmHg (140/90). However, BP increases with age and hence only elderly people over 60 years with BPs above 150/90 mmHg may require treatment [41]. Among the physiological mechanisms of hypertension, the renin-angiotensin system has attracted much scientific attention. Renin and angiotensin-converting enzyme (ACE) are the main enzymes involved in the renin-angiotensin system (RAS) [112].

Many synthetic antihypertensive drugs have been reported to cause side effects such as dizziness, dysgeusia, headache, angioedema, and cough [41]. Thus, the search for antihypertensive biopeptides from foods has increased (Table 1). Food-derived antihypertensive peptides are known for their high tissue affinities and hence may be more slowly eliminated from tissues compared to synthetic drugs [5]. To release antihypertensive peptides from whey, we fermented whey from bovine milk with several *Lactobacillus* species and found that *Lactobacillus helvelticus* fermented whey hydrolysates contained peptides AQSAP, IPAVF, APLRV and AHKAL which showed strong angiotensin 1-converting enzyme inhibition. These peptides, at least in part, contributed to the ACE inhibitory effect of the fermentate. Whey fermented with *Lactobacillus brevis* also contained a potent ACE inhibitory peptide identified as AEKTK [38]. Two tripeptides, VPP and IPP from casein have been shown to significantly reduce high blood pressure in humans [113]. The peptides were first reported by Nakamura et al. [114] when they fermented β-casein using *Saccharomyces cerevisiae* and *Lactobacillus helveticus* CP790. Many other food derived antihypertensive peptides have been shown to effectively reduce high blood pressure after a single dose [72,115,116] and after long term administrations [117,118,119] in animal models.

Recently, a potent ACE inhibitory peptide DPYKLRP was isolated from lactoferrin after *Kluyveromyces marxianus* fermentation. The peptide (10 mg/kg body weight) reduced systolic blood pressure (SBP) in spontaneous hypertensive rats (SHRs) by 27 mmHg relative to control rats which received 650 μL of saline [21]. Also, the dipeptide DY in aqueous extracts from bamboo shoots has been shown to reduce SBP by 18 mmHg in SHRs when administered at 10 mg/kg body weight/day [120]. In another study, Fitzgerald et al. [121] isolated the peptide IRLIIVLMPILMA from hydrolyzed *Palmaria palmata* with papain. The peptide reduced systolic blood pressure by 33 mmHg in SHR. In a double-blind parallel group intervention study, 89 hypertensive subjects who consumed fermented milk containing 5 mg IPP and VPP daily for 12 weeks and a high dose (50 mg/day) for the next 12 weeks experienced a reduction in arterial stiffness leading to reduced blood pressure [122]. Similarly, a spread containing 4.2 mg IPP and VPP significantly reduced SBP by −4.1 mmHg in 104 middle-aged hypertensive subjects after 10 weeks of consumption [123]. Conversely, consumption of a fermented milk containing 5 mg of the tripeptides did not affect the BP of subjects with metabolic syndrome [124]. Over the years, milk-derived antihypertensive peptides are the most studied and several reviews concerning their production, bioavailability and incorporation into foods have been published [125,126,127]. Also, several fermented milk products such as Evolus^®^ (Valio Ltd., Helsinki, Finland), Danaten^®^, Ameal^®^ and Calpis^®^ (Calpis Co., Tokyo, Japan) [128] have been developed for managing high blood pressure.

However, the European food safety authority does not consider that a proof of cause and effect relationship between the consumption of these foods has been established [129].

For a strong antihypertensive activity, the position of certain amino acid residues is critical. For instance, the presence of branched amino acids such as valine and isoleucine are important for ACE inhibition [114]. Therefore, hydrolyzing proteins with thermolysin will increase the chances of generating peptides with terminal branched chain amino acids. Also, the presence of proline at the *C*-terminal has also been shown to enhance ACE inhibition [114] and hence hydrolyzing proteins with prolyl endopeptidases and other proteases that generate proline containing peptides may be helpful in producing antihypertensive peptides. Most of the studies that examined the role of peptide chain amino acid position on the potency of ACE inhibition involved statistical modeling coupled with in vitro experiments and they all agree that the *C*-terminal sequence are important for ACE inhibition. Yet there is still contradiction about which specific amino acids must be present to enhance *C*-terminal activity [130].

Several mechanisms may account for the inhibitory ability of antihypertensive peptides. The peptides may inhibit ACE activity by binding to the enzyme. ACE cleaves a dipeptide from angiotensin-I to yield angiotensin II (a vasoactive peptide) which binds with receptors on the vascular wall to cause blood vessel contractions [21]. Therefore, inhibition of ACE reduces high blood pressure. Other peptides such as IRLIIVLMPILMA inhibit renin [121], an enzyme that cleaves a dipeptide from angiotensinogen to yield angiotensin I [131]. Some antihypertensive peptides enhance nitric oxide production [132,133] while other peptides including RVPSL block angiotensin II receptors [134]. IPP has also been proposed to enhance Ang-(1–7) binding with Mas receptors and promotes bradykinin-mediated vasorelaxation which attenuate the development of hypertension [135].

Meanwhile, though many antihypertensive peptides are known and their activities have been confirmed in animal studies, more human intervention studies that use non-invasive techniques to measure hypertension parameters are required to establish the health effects of the peptides [129].

### 3.4. Anti-Cancer

Most synthetic anticancer agents have been associated with nephrotoxic [136,137], neurotoxic, cardiotoxic [138] and gonadotoxic [139,140] side effects. For this reason, the search for anti-cancer bioactive peptides from food sources has increased.

A cell selective peptide HVLSRAPR isolated from *S. platensis* hydrolysates showed strong inhibitory activity against HT-29 cancer cell proliferation but showed little inhibition against normal liver cells [141]. In another study, the tripeptide WPP isolated from blood clam muscle showed strong cytotoxicity toward PC-3, DU-145, H-1299 and HeLa cell lines [95]. Two peptides from tuna cooking juice KPEGMDPPLSEPEDRRDGAAGPK and KLPPLLLAKLLMSGKLLAEPCTGR have been shown to exhibit strong antiproliferative activity in breast cancer cell line MCF-7. The peptides induced cell arrest in the S phase by increasing p21 and p27 expression while decreasing cyclin A expression. Additionally, the peptides cleaved caspase 3, downregulated Bcl-2, PARP and caspase 9 expression but upregulated p53 and Bax expression [142]. Sepia ink protein hydrolysates contained a peptide QPK which significantly inhibited the proliferation of DU-145, PC-3 and LNCaP cells. The peptide also decreased the expression of the anti-apoptotic protein Bcl-2 and increased the expression of apoptogenic protein Bax [143].

In the same way, the peptide LANAK, isolated from oyster hydrolysate showed anticancer activity against human colon carcinoma (HT-29) cell lines [87]. Another peptide RQSHFANAQP from chickpea hydrolysate increased the level of p53 in breast cancer cell lines and inhibited their proliferation [144] while YALPAH from *Setipinna taty* induced apoptosis in prostate cancer PC-3 cells [145]. Soybean protein hydrolysates contain many anticancer peptides such as Lunasin, RKQLQGVN [146], GLTSK, LSGNK, GEGSGA, MPACGSS and MTEEY [147]. These peptides have been reported to exert strong antiproliferative effects on colorectal cancer HT-29 cells. The peptides RHPFDGPLLPPGD, RCGVNAFLPKSYLVHFGWKLLFHFD and KPEEVGGAGDRWTC obtained from *Dendrobium catenatum* Lindley demonstrated strong antiproliferative activity against HepG-2, SGC-7901 and MCF-7 cancer cells [148]. Peptides from rapeseed protein fermentates have also been shown to inhibit the proliferation of human HepG2 liver cancer, human MCF-7 breast cancer and human MCF-7 breast cancer cell lines [149].

Over the years, only a few studies have tested the potential cytotoxity of anticancer peptides against normal cells. However, more of such studies are needed to confirm the safety of food derived anticancer peptides. To date, most of the anticancer ability of bioactive peptides have only been assessed in vitro while very little is known about the in vivo activities of such peptides. Studies on the anti-cancer bioactive peptides in animal models are therefore warranted.

### 3.5. Antimicrobial Peptides

Antimicrobial peptides (AMP) are known to exert direct effects on a wide range of bacteria, yeast and viruses. Interestingly, many antimicrobial peptides show additional bioactivities such as antioxidant activities [150], immunomodulation [151], and wound healing activity [152]. These properties of AMPs make them better alternatives for conventional antibiotics which have recorded much resistance among pathogenic bacteria. AMPs vary in length (between 12–50 amino acids), amino acid composition, charge and position of disulphide bonds [153]. The presence of a positive charge or the presence of both hydrophilic and hydrophobic amino acids at the terminals (amphipathic) are recognized as major structural motifs by which AMPs interact with microbes. It has been shown that the antimicrobial potency of a cationic AMP is directly related to the product of its charge, hydrophobicity and length of the peptide [153]. AMPs may directly kill bacteria either by making pores through the bacteria cell membrane [154,155] or by interacting with macromolecules inside the microbial cells [156,157]. It has been reported that AMPs rich in positively charged amino acids such as arginine and lysine enter into cells by inducing energy dependent endocytic pathway such as micropinocytosis [158]. Many AMPs have been identified over the years and are available on several databases such as APD3 [159], CAMPR3 [160], DRAMP [161] and YADAMP [162]. Extensive studies have been done on the antimicrobial activity of milk derived peptides and AMPs have been mostly identified in peptides fragments from casein, and β-lactoglobulin and α-lactalbumin [162]. Recently, Zhang et al. [155] found that the peptide ELLLNPTHQIYPVTQPLAPV isolated from human colostrum killed bacteria by cell wall and cytoplasmic membrane destruction. In another study, an AMP SSSEESII from α_s2_–casein was found to inhibit the growth of *Listeria innocua, Micrococcus luteus, Salmonella enteritidis* as well as *E. coli*. The nanopeptide, IKHQGLPQE in casein hydrolysates effectively reduced the number of pathogenic bacteria spiked in infant formula [163]. Potent AMPs have also been isolated from fish and fish products. Hydrolysis of Mackerel by-products yielded SIFIQRFTT which inhibited *Listeria innocua* and *Escherichia coli* [164]. When anchovy cooking waste water was treated with protamix, the AMP GLSRLFTALK was isolated. The peptide showed strong inhibition against *S. aureus*, *B. subtilis, S. pneumoniae, E. coli*, *S. dysenteriae, P. aeruginosa* and *S. typhimurium* [165]. Hydrolysis of *Scorpaena notata* (Small red scorpionfish) viscera protein with a neutral protease from *Trichoderma harzianum* showed remarkable antimicrobial activities. The peptide FPIGMGHGSRPA was isolate from the hydrolysate and was found to inhibit *Bacillus subtilis, Bacillus cereus, Listeria innocua, Salmonella* sp. and *E.* coli [166]. In addition to AMPs from fish and fish products, AMPs were identified in bovine blood. The peptide VNFKLLSHSLLVTLASHL isolated from bovine haemoglobin strongly inhibited the growth of *Candida albicans*, *Escherichia coli* and *Staphylococcus aureus* [167]. Controlled pepsin hydrolysis of haemoglobin yielded the AMPs VLSAADKGNVKAAWGKVGGHAAEYGAEALERMF, ASHLPSDFTPAVHASLDKFLANVSTVLTSKYR and VLSAADKGNVKAAWGKVGGHAAEYGAEALERMFLSF. The peptides showed strong inhibition against *Salmonella enteritidis, Escherichia coli, Shigella sonnei, Micrococcus luteus, Enterococcus faecalis, Listeria innocua, Staphylococcus saprophyticus, Bacillus cereus* and *Staphylococcus simulants* [168,169].

### 3.6. Multifunctional Peptides

Food-derived bioactive peptides with single activities have been well documented, yet only few peptides with multiple functions have been reported. Meanwhile, single peptides with multifunctional bioactivities will be more preferred over single activity peptides as the former would simultaneously elicit multiple health benefits. For this reason, García-Mora et al. [170] hydrolyzed lentil proteins using Savinase^®^ (Novozymes, Bagsvaerd, Denmark) to search for peptides with multiple functions. They observed that peptides LLSGTQNQPSFLSGF, NSLTLPILRYL and TLEPNSVFLPVLLH present in the hydrolysate had strong antioxidant and antihypertensive effects. YSK from rice bran protein [4], WVYY and PSLPA from hemp seed protein hydrolysates [171] also showed both antioxidant and antihypertensive effects. Cummin seed peptides CSP1, CSP2 and CSP3 have been reported to exhibit cholesterol lowering activity [105], anti-oxidant and anti-amylase activities in vitro [99]. Four other peptides, YINQMPQKSRE, YINQMPQKSREA, VTGRFAGHPAAQ and YIEAVNKVSPRAGQF isolated from egg yolk have been reported to show antidiabetic, ACE inhibitory and antioxidant activities [172]. These peptides could be important in managing metabolic diseases such as diabetes and hypercholesterolemia.

Another peptide WPP isolated from *Tegillarca granosa* hydrolysates is reported to have strong antioxidant and anticancer activities [95]. Also, RQSHFANAQP peptide from chickpea albumin showed strong antioxidative and anticancer effects in MCF-7 and MDA-MB-231 cells [144].

A peptide that may prevent reactive oxygen species-induced cancer has been isolated recently. The polypeptide was obtained from *Pleurotus eryngii* mycelium and has been reported to possess a strong reducing power and a strong oxygen radical scavenging ability. The peptide also showed strong antitumor effect against stomach, cervical and breast cancer cell lines and stimulated the immune system [173]. Another recent study has shown that lunasin isolated from quinoa has strong anti-inflammatory and antioxidant abilities [174]. Since reactive oxygen species can cause inflammation [175], lunasin could be important in treating or preventing inflammatory diseases. In a similar way, NTDGSTDYGILQINSR from egg white lysozyme hydrolysates scavenge DPPH and ABTS radicals and also inhibits the growth of both Gram-negative and Gram-positive bacteria [150]. The functions of this peptide make it a potential agent for food preservation.

Low molecular weight peptides from various foods have also shown important multifunctional abilities. For instance, Aguilar-Toalá et al. [7] fermented milk with *Lactobacillus plantarum* strains and observed that the crude fermented milk extracts had strong antihemolytic, antioxidant, anti-inflammatory, antimicrobial and antimutagenic activities. Also, low molecular weight peptides from hydrolyzed brewer’s yeast showed strong antiulcer and antiproliferative activity against leukemia cell lines [176]. Low molecular weight peptides from *I. badionotus* hydrolysates have also been reported to inhibit ACE activity, kill colorectal cancer cells and scavenge free radicals [177]. Similarly, peptides in cowpea hydrolysates were found to exhibit antioxidant activities and inhibit HMGCoA reductase activity [109]. Likewise, Rohu roe hydrolysates showed strong ACE inhibitory ability as well as antiproliferative activity against Caco-2 cell lines [178].

These observations could however be due the different bioactive peptides released during the fermentation and hydrolysis process and not necessarily the presence of single multifunctional peptides. Though the isolation of single multifunctional peptides from the hydrolysates would be of much interest, consumption of the hydrolysates may yield multiple health effects.

## 4. Effects of Processing Methods on Bioactivity

Food processing methods can significantly affect the biological activity of bioactive peptides. Physical processing methods such as ultrasound, heat and irradiation may affect the protein structure and functions. Processing could also result in maillard reactions and lead to the production of allergenic compounds [179]. Processing may increase the susceptibility of peptides to gastrointestinal digestion, absorption and response to the immune system. Therefore, it is important to determine the optimal conditions within which proteins (and peptides) could be processed to maintain or enhance their bioactivities. However, processing methods that may reduce the activity of one peptide may enhance the activities of others. For instance, the antibacterial activity of α-lactalbumin [180] and lysozyme [181] increased after they were denatured by heat while other peptides may lose their activity after heating.

The effects of boiling on the activity of biopeptides may depend on the enzyme formulation as well as the treatment conditions of the parent protein. It has been shown that hydrolyzing raw casein using chymotrypsin yielded antidiabetic peptides with higher bioactivity that those release from boiled casein [182] and hence, raw milk hydrolysates may be more effective in diabetes management than pasteurized milk. Similarly, thermal processing has been reported to significantly decrease the antioxidant activity of cowpea derived peptides. However, the thermal treatment increased the ability of the peptides to inhibit the micellar solubility of cholesterol relative to raw samples [109].

In another study, though the DPP-IV, *α*-glucosidase inhibitory activities and ACE inhibitory potencies of Navy beans hydrolysates did not change significantly after cooking, *α*-amylase inhibitory ability reduced significantly [183]. ACE inhibitory peptides tend to retain their bioactivity after thermal processing. For instance, the ACE inhibitory peptides YAGGS and YAAGS from beans maintained their bioactivities even after precooking, and this indicates that precooked beans could still provide beneficial health effects [183]. The peptides KAAAAP, AAPLAP, KPVAAP, IAGRP, and KAAAATP from ham also retained their ACE inhibitory abilities after heat treatment (at 117 °C for 6 min) [184]. Similarly, collagen derived ACE inhibitory peptides retained their bioactivity between a pH range of 2–6 and after 2 h of heating at 100 °C. Their bioactivity however significantly reduced in alkaline conditions [185].

In another study, pulsed electric field (PEF) was used to improve the ability of antioxidant peptides KCHQP (from pine nut) [186] and SHCMN (from soybean protein) [187]. Though PEF did not change the basic structures of the peptides, their zwitter potentials were significantly reduced. It is therefore important to ascertain the optimal conditions within which bioactive peptides could be processed to retain their bioactivity.

## 5. Challenges in Bioactive Peptide Development

Since bioactive peptides are encrypted in foods, the extent of protein hydrolysis during their production is an indispensable factor worth considering. Yet, peptides released during fermentation and enzymatic proteolysis remain susceptible to further hydrolysis as long as the enzymatic reaction or the fermentation process goes on. Such a situation may result in a decrease or loss of bioactivity due to continuous degradation of the peptides. This makes designing of a kinetic model for protein hydrolysis very challenging [180].

Also, peptides produced by microbial fermentation using wild microbes are not reproducible. This is because, the microbes are live cells and their metabolic activities, the type of enzymes and enzyme levels cannot be controlled. Therefore, the quantities of specific bioactive peptides released after fermentation cannot be guaranteed. Therefore, improved strains or genetically recombinant strains or pure enzymes may help mitigate this challenge. Meanwhile, the use of enzymes for protein hydrolysis is more expensive than microbial fermentation. Also, bioactive peptides in food hydrolysates sometimes have improved activity due to their synergistic effects with other components in the hydrolysate. Therefore, some single isolated peptides may demonstrate reduced bioactivities when tested alone as nutraceuticals.

Another important challenge in bioactive peptide production is the stability of the generated peptides. Most food derived peptides are easily degraded in the gut and therefore do no exhibit any resultant activity in the body when tested in vivo. In this case, pure isolated peptides with good activities could be stabilized against digestion by inserting a structure inducing probe tail [188] and also by clipping the peptide sequence. Such stabilization strategies would improve the bioavailability of the peptides after consumption.

## 6. Conclusions and Future Perspectives

Bioactive peptides from foods are valuable functional agents in healthy diets that can prevent and treat diseases. Consumer awareness of the effects of functional foods on health is a strong drive for the search and production of bioactive peptides in foods. Milk derived tripeptides IPP and VPP are the most studied food derived antihypertensive peptides and have shown positive effects in human studies. This warrants the confirmation of other food derived bioactive peptides in human studies. Many food hydrolysates have also shown multifunctional bioactive effects [7,176,177], however, their components are unknown. Identifying the peptides in such hydrolysates will be important in studying the mechanisms by which they exert their health effects.

The search of bioactive peptides through microbial fermentation will remain a promising and a cheap strategy for generating bioactive peptides in foods as generally regarded as safe microbial proteolytic systems yield several peptides of diverse potentials during fermentation. In the near future, development and use of genetically improved strains will become important as they would release large amounts of proteolytic enzymes to hydrolyze food proteins. Also, pure food derived bioactive peptides would soon be abundant on the market and sold as nutraceuticals. Such peptides could be regulated as drugs since they would be well characterized and their properties and mechanisms of action established.

## Figures and Tables

**Table 1:** Bioactive peptides and their functions.

Peptide	Function	Reference
**Antimicrobial**		
LRLKKYKVPQL	Interacts with bacteria to cause inhibition.	[61]
PGTAVFK	Causes bacteria and yeast membrane destruction.	[62]
KVGIN, KVAGT, VRT, PGDL, LPMH, EKF, IRL	Inhibits *Listeria ivanovii* and *E. coli* growth.	[63]
Lp-Def_1_	Interacts with and impairs mitochondrial functions in *C. albicans*.	[64]
Maize α-hairpinins	Binds to microbial DNA to cause cell death.	[65]
**Antihypertensive**		
DVWY, FQ, VVG. DVWY, VAE, WTFR	Inhibit ACE in thoracic aorta tissue and suppress angiotensin II-mediated vasoconstriction.	[5,66]
DPYKLRP, PYKLRP, YKLRP, GILRP		[21]
VPP, IPP	Competitively bind and inhibit ACE and results in blood pressure reduction	[44]
GAAGGAF		[67]
LIVTQ, LIVT		[68]
LLKPY		[69]
AHLL		[70]
FISNHAY		[71]
AAATP		[72]
LGL, SFVTT		[73]
IIT		[74]
ADVFNPR, VVLYK, LPILR, VIGPR	Lower endothelia-1 levels significantly	[75]
**Anti-type 2 diabetes mellitus**		
PPL	Inhibits dipeptidyl peptidase-IV	[74]
YP, LP, IPI, VPL, IPA, IPAVF		[33]
PGVGGPLGPIGPCTE, CAYNTERPVDRIR, PACCGPTISRPG		[76]
GPAE, GPGA		[77]
MHQPPQPL, AWPQYL, SPTVMFPPQSVL, VMFPPQSVL, AWPQYL and INNQFLPYPY		[78]
ILAP, LLAP, MAGVAHI		[79]
IP, MP, VP, LP		[80]
LKPTPEGDL, LPYPY, IPIQY and WR		[81]
**Immunomodulatory**		
GFLRRIRPKLKT	Significantly inhibits LPS-induced nuclear translocation of NF-κB/p65, inhibits IL-1β and enhances TNF-α release.	[82]
St20	Inhibits human T lymphocyte surface marker CD69 expression and cytokine IL-2 secretion. St20 also inhibits TNF-α and IFN-γ secretion in the activated human T lymphocytes.	[83]
PTGADY	Significantly increases the production of IL-2, IL-4, and IL-6.	[84]
**Anti-oxidation**		
IP, MP, VP, LP	Scavenge Hydroxyl radicals	[80]
AEERYP, DEDTQAMP	Scavenge reactive oxygen species	[32]
DHTKE, MPDAHL, FFGFN	Oxygen radical scavenging, DPPH radical scavenging.	[85]
RPNYTDA, TSQLLSDQ, TRTGDPFF, NFHPQ	DPPH and ABTS radical scavenging, FRAP-Fe^3+^ reducing ability.	[86]
LANAK, PSLVGRPPVGKLTL, VKVLLEHPVL	DPPH radicals scavenging ability	[87]
WEGPK, GPP and GVPLT	DPPH, ABTS, and hydroxyl radicals scavenging ability, inhibiting lipid peroxidation	[88]
PYSFK, GFGPEL, GGRP		[89]
LSGYGP	Hydroxyl radicals scavenging.	[90]
GSGGL, GPGGFI, FIGP	DPPH, Hydroxyl and reactive oxygen radical scavenging	[89]
PIIVYWK, TTANIEDRR, FSVVPSPK	Hydrogen peroxide radicals scavenging	[91]
YYIVS		[92]
FIMGPY, GPAGDY and IVAGPQ	DPPH, Hydroxyl and reactive oxygen radical scavenging	[93]
ATSHH	DPPH radicals scavenging	[94]
TPP	Lipid peroxidation, radical scavenging activity.	[95]
WVAPLK	DPPH, Hydroxyl and reactive oxygen radical scavenging	[96]
GASRHWYFL	DPPH, superoxide, ABTS and hydroxyl radical scavenging, lipid peroxidation.	[97]
PYSFK, GFGPEL, VGGRP		[98]

A = alanine, R = arginine, N = asparagine, D = aspartic acid, C = cysteine, E = glutamic acid, Q = glutamine, G = glycine, H = histidine, I = isoleucine, L = leucine, K = lysine, M = methionine, F = phenylalanine, P = proline, S = serine, T = threonine, W = tryptophan, Y = tyrosine, V = valine. LDL: Low-density lipoprotein, IL: Interleukin, TNFα: tumor necrosis factor alpha, DPPH: 2,2-diphenyl-1-picrylhydrazyl, ABTS: 2,2′-azinobis-(3-ethylbenzothiazoline-6-sulfonic acid), FRAP: ferric reducing antioxidant power, IFNγ: interferon gamma.
